# Bioremediation of Synthetic and Industrial Effluents by *Aspergillus niger* Isolated from Contaminated Soil Following a Sequential Strategy

**DOI:** 10.3390/molecules22122244

**Published:** 2017-12-16

**Authors:** Tahsin Gulzar, Tayyaba Huma, Fatima Jalal, Sarosh Iqbal, Shazia Abrar, Shumaila Kiran, Sofia Nosheen, Waqar Hussain, Muhammad Asim Rafique

**Affiliations:** 1 Department of Applied Chemistry & Biochemistry, Government College University Faisalabad, Faisalabad 38000, Pakistan; tahsingulzar1@yahoo.com (T.G.); sarosh.iqbal@yahoo.com (S.I.); shaziaabrar@gcuf.edu.pk (S.A.); waqar1214@gmail.com (W.H.); 2 Department of Biotechnology and Bioinformatics, Government College University Faisalabad, Faisalabad 38000, Pakistan; tayyabahuma@gcuf.edu.pk; 3 Department of Zoology, Government College University Faisalabad, Faisalabad 38000, Pakistan; fatima_jalal@hotmail.com; 4 Department of Environmental Science, Lahore College for Women University, Lahore 58000, Pakistan; 5 Department of Public Administration, Government College University Faisalabad, Faisalabad 38000, Pakistan; asimchoudhary786@gmail.com

**Keywords:** *Aspergillus niger* (*A. niger*) isolation, Fenton treatment, biological treatment, decolorization, ligninolytic enzymes, sequential treatment, mineralization study

## Abstract

The present study aimed to assess and compare the ability to remediate synthetic textile and industrial wastewaters by Fenton treatment, a biological system and sequential treatments using *Aspergillus niger* (*A. niger*). All studied treatments were found to be effective in decolorization of the effluents under study. Fenton treatment followed by *A. niger* showed excellent potential for the maximum decolorization of the synthetic and industrial effluents under study. The effectiveness of sequential treatment was evaluated by water quality parameters such as total organic carbon (TOC), Biological Oxygen Demand (BOD5) and Chemical Oxygen Demand (COD) before and after each treatment. The results indicated that *A. niger* is an effective candidate for detoxification of textile wastewaters.

## 1. Introduction

Pollution problems due to textile industry effluents have increased in recent years. Approximately 75% of the dyes discharged by textile processing industries belong to the classes of reactive (36%), acid (25%) and direct (15%) dyes. Approximately 50% of the applied dye is lost in the effluent during the textile dyeing process [1]. Recent studies have shown that azo dyes contribute to the mutagenic activity of ground and surface waters polluted by textile effluents [2]. Furthermore, their discharge into surface water leads to aesthetic problems and obstructs light penetration and oxygen transfer into water bodies, hence affecting aquatic life [3]. Moreover; it is very difficult to treat textile industry effluents because of their high BOD, COD, heat, color, pH and the presence of metal ions [4]. Therefore, treatment of industrial effluents containing azo dyes before its discharge into wastewater bodies is deemed necessary. As the characteristics of dye wastewaters are very variable, many different physical, chemical and biological treatment methods are in use for their treatment, and which one is effective depends upon the type of dye wastewater [5]. Interest is now focused on the microbial biodegradation of dyes as a better alternative which offers distinct advantages over conventional modes of treatment. This method is more economical and leads to less accumulation of relatively harmless sludge [6]. A long time can be required for the disappearance of the active dye molecule and its complete mineralization during biodecolorization. The integration of two processes, photocatalysis and biological treatment can be helpful to reduce the costs [7]. Many studies have reported the use of combination methods for the removal of dyes and colorants from wastewaters and they have been proposed to be the ideal solution for bioremediation of wastewaters [8,9]. Advanced oxidation processes are used as a pre-treatment step for the enhancement of the biodegradability of wastewater comprising recalcitrant compounds [10]. Keeping in mind the hazardous nature of dyes and their possible detoxification, the present study was planned, which involves the isolation of *Aspergillus niger* (*A. niger*) from local contaminated soil and studying its role in the mineralization of synthetic and industrial textile effluents used in local textile industries by means of an integrated process involving Fenton catalysis followed by biological treatment.

## 2. Results and Discussion

### 2.1. Fenton Treatment

**Figure 1:**
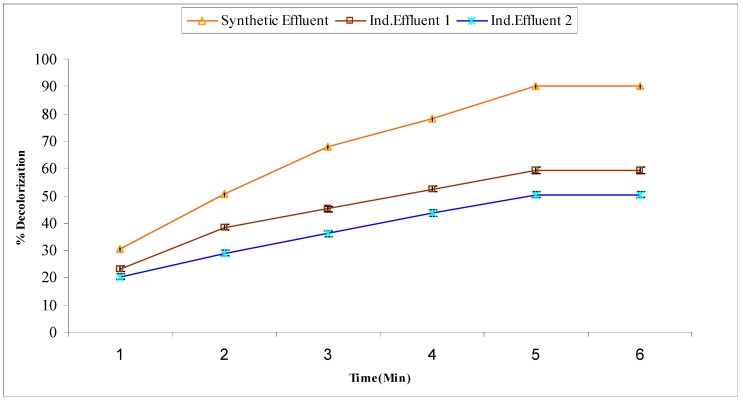
Decolorization (%) of synthetic and industrial effluents by Fenton treatment.

Fenton treatment was used for the decolorization of synthetic and industrial wastewaters. The mechanism involved is based on the hydroxyl radicals generated from H_2_O_2_ in the presence of Fe^+2^ ions. The experiments were performed under pre-optimized conditions like temperature 50 °C, pH 3.5, 80 mg of FeSO_4_·7H_2_O, and a reaction time of one hour. In the Fenton process for synthetic effluent, the decolorization (%) was found to be 30.42, 50.56, 67.71, 78.26, 90.31 and 90.31% at 10, 20, 30, 40, 50 and 60 min, respectively. The results of decolorization (%) for industrial effluent 1 were 23.22, 38.43, 45.12, 52.45, 59.32, and 59.32% at 10, 20, 30, 40, 50 and 60 min. Similarly, industrial effluent 2 showed a rate of decolorization (%) of 20.36, 28.93, 36.12, 43.67, 50.32, and 50.32% at 10, 20, 30, 40, 50 and 60 min (Figure 1). Comparison among results indicated the maximum decolorization (%) of synthetic effluent at 50 min that was 90.31% and for industrial effluents 1 and 2 it was 59.32% and 50.32%, respectively.

Several studies have reported that the use of iron-containing oxides as catalysts in Fenton reactions has advantages of low cost and easy operation, and may exhibit excellent catalysis performance in the removal of organic contaminants [11,12]. The Fenton process/a combination of UV with Fenton or Fenton-like conditions, are found to be more efficient and less pH-dependent treatment methods [13]. This has significant advantages over classic homogenous Fenton catalysis, such as no pH restriction, no iron sludge formation and easy separation of the catalyst from the treated wastewater [14,15].

### 2.2. Biological Treatment

#### 2.2.1. Decolorization of Synthetic and Industrial Effluents by *A. niger*

**Figure 2:**
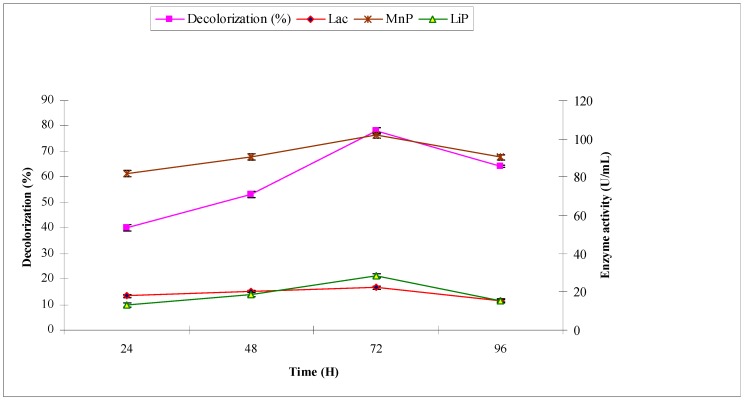
Effect of reaction time on biodecolorization of synthetic effluent and activities of ligninolytic enzymes of *A. niger*.

**Figure 3:**
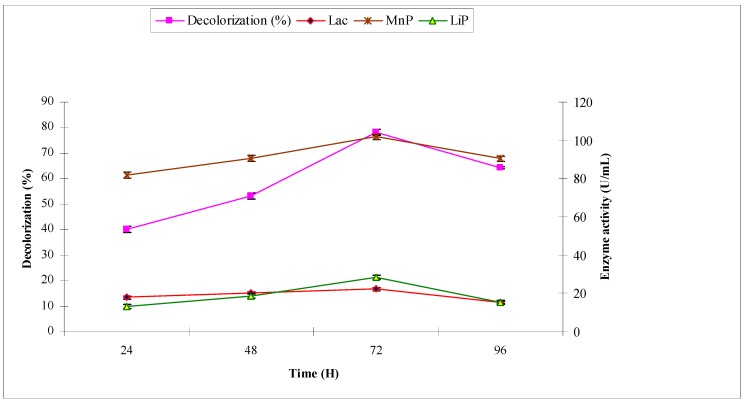
Effect of reaction time on biodecolorization of industrial effluent 1 and activities of ligninolytic enzymes of *A. niger*.

The synthetic effluent was subjected to treat with *A. niger* under pre-optimized conditions. The purpose was to investigate the decolorization (%) and production of ligninolytic enzymes laccase, manganese peroxidase and lignin peroxidase, which have potential to break down the chromophoric groups of synthetic dyes. *A. niger* decolorized the synthetic effluent by 40.2, 53.2, 78.2 and 64.3 at 24, 48, 72 and 96 h, respectively. The maximum decolorization of 78.2% was observed at 72 h with maximum ligninolytic enzyme production (in U/mL) of 16.57, 101.79 and 28.45 of laccase, manganese peroxidase and lignin peroxidase, respectively (Figure 2). When industrial effluent 1 was subjected to bio-treatment using *A. niger*, the maximum decolorization of 52.2% was observed at 72 h with maximum ligninolytic enzyme production (U/mL) of 16.52, 60.87 and 25.43 of laccase, manganese peroxidase and lignin peroxidase, respectively (Figure 3).

**Figure 4:**
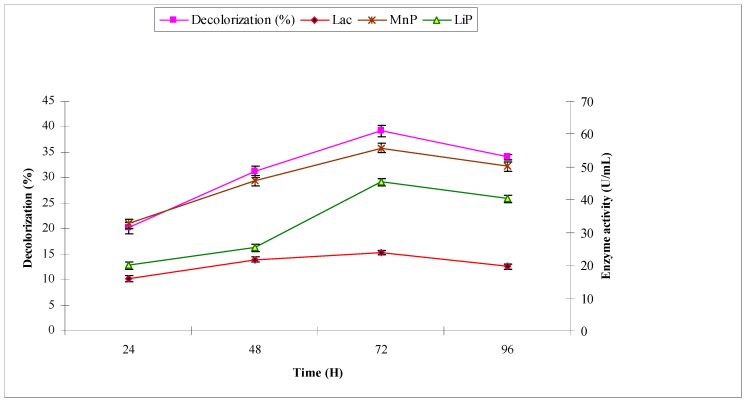
Effect of reaction time on biodecolorization of industrial effluent 2 and activities of ligninolytic enzymes of *A. niger*.

With industrial effluent 2, the maximum decolorization (39.2%) was observed at 72 h with maximum ligninolytic enzymes production (in U/mL) of laccase 15.34, manganese peroxidase 55.81 and lignin peroxidase 45.39, respectively (Figure 4). Manganese peroxidase production was higher in the current study than other two ligninolytic enzymes.

Researchers have demonstrated that *A. niger* could efficiently decolorize different kinds of dyes like monoazo dyes (Reactive Red 198 and Reactive Orange 122), a diazo dye (Reactive Yellow 160), a phthalocyanine dye (Reactive Blue 21) and an anthroquinone dye (Reactive Blue 19) with differences in the decolorization ability which depend upon concentration of the dye solution [16,17]. *A. niger* could decolorize dyes in relatively wide range of pH, this makes the fungus suitable for practical treatment of dyes [18]. Our literature survey showed the production of ligninolytic enzymes i.e., laccase, MnP, LiP by *P. chrysosporium* and *A. niger* and their subsequent use for decolorization of distillery effluents [19,20,21]. Researchers have reported *A. niger* MT-1 as a suitable bio-accumulator for removal of Reactive Black-5 dye in effluents produced by textile industries. They concluded that the biomass concentration and bioaccumulation efficiency of the fungal mycelium were correlated with each other. The time required for decolorization increased with the increase in dye concentration [22,23]. Researchers reported the involvement of ligninoilytic enzymes of *Aspergillus* sp. in the degradation of azo dyes in textiles wastewaters [19]. Soil fungi possess ligninolytic enzymes and play an important role in the degradation of lignocellulose in soil ecosystems. These lignin-degrading enzymes are directly involved not only in the degradation of lignin in their natural lignocellulosic substrates but also in the degradation of various xenobiotic compounds, including dyes [24,25,26,27].

### 2.3. Sequential Treatment

Sequential treatment study was done to evaluate the efficiency of treatment methods either they are more effective when they are treated alone or in combination with each other. Sequential treatment was done via two different ways.

#### 2.3.1. Fenton Treatment Followed by Biological Treatment

**Figure 5:**
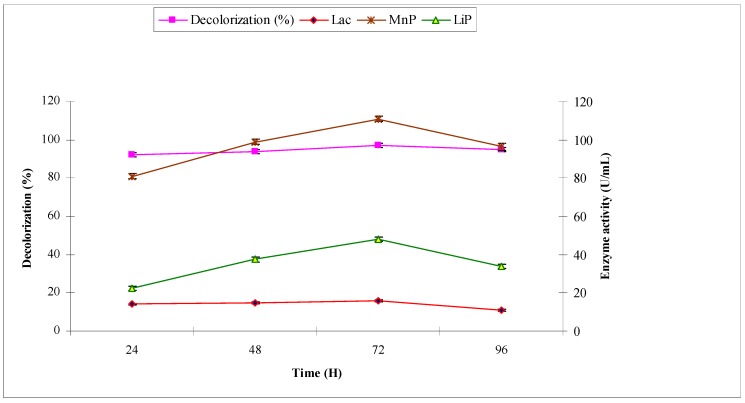
Effect of reaction time on biodecolorization of synthetic effluent and activities of ligninolytic enzymes of *A. niger* by Fenton treatment followed by biological treatment.

**Figure 6:**
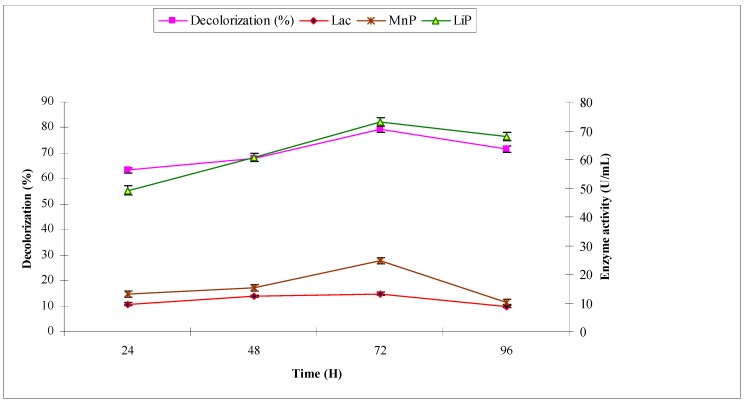
Effect of reaction time on biodecolorization of industrial effluent 1 and activities of ligninolytic enzymes of *A. niger* by Fenton treatment followed by biological treatment.

**Figure 7:**
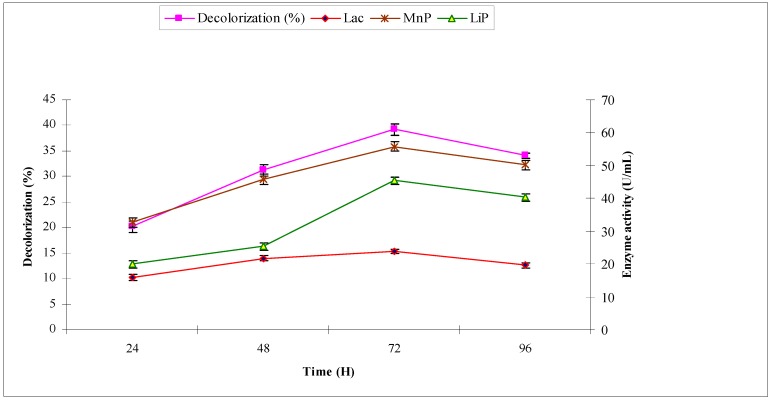
Effect of reaction time on biodecolorization of industrial effluent 2 and activities of ligninolytic enzymes of *A. niger* by Fenton treatment followed by biological treatment.

This sequential treatment was done using pre-optimized conditions. For this purpose, each effluent was subjected to Fenton treatment under the previously optimized experimental conditions (pH 3.5, H_2_O_2_ 1 × 10^−2^ M, FeSO_4_ 3.5 × 10^−5^ M and temperature 50 °C). The Fenton-treated effluents were then subjected to biodecolorization by *A. niger* under the pre-optimized growth conditions (pH 4.5, temperature 30 °C, inoculum size 4 mL, rice bran 1.5 g, MnSO_4_ 1 mM). Decolorization (%) increased as time was increased from 24 h to 72 h (97.2%). The decolorization (%) was decreased by further increases in time. The maximum decolorization (97.2%) was observed at 72 h with maximum ligninolytic enzyme production (U/mL) of 15.99, 110.97 and 47.94 for laccase, manganese peroxidase and lignin peroxidase, respectively (Figure 5). For industrial effluent 1, observed decolorization (%) was 65.9, 73.9, 80.5 and 75.8% at 24, 48, 72 and 96 h, respectively (Figure 6). The industrial effluent 2 was decolorized maximum at a time of 72 h. Decolorization (60.5%) was observed at 72 h with maximum ligninolytic enzymes production (U/mL) 14.91, 68.81 and 25.02 of laccase, manganese peroxidase and lignin peroxidase, respectively (Figure 7). Sequential process for mineralization of dyes showed a good potential towards decolorization (up to 97%) as well as mineralization (up to 90%) of synthetic azo dyes [28].

Based on the available literature, it can be concluded that the microbial decolorization of azo dyes is more effective in sequential microaerophilic/aerobic and aerobic/microaerophilic processes [29,30]. Sequential microaerophilic/aerobic processes of azo dyes degradation represent a promising approach for detoxification of textile effluents [31].

#### 2.3.2. Biological Treatment Followed by Fenton Treatment

**Figure 8:**
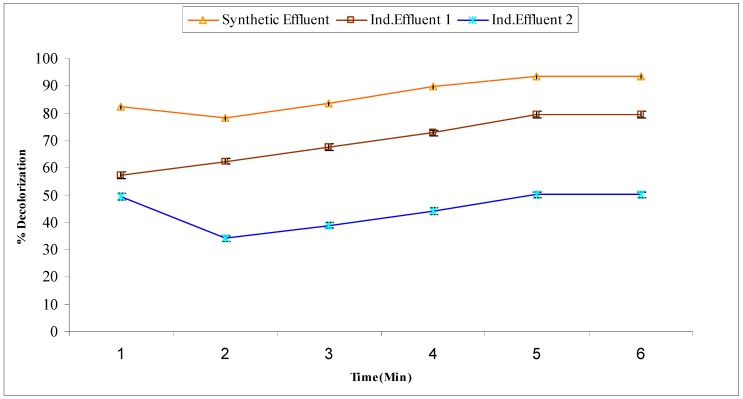
Decolorization (%) of *A. niger (AN)* treated effluents by Fenton treatment.

In sequential treatment, the samples treated with *A. niger* were subjected to Fenton treatment under pre-optimized conditions. The decolorization (%) observed was 82.26, 80.37, 83.48, 89.67, 93.95 and 93.95% at 10, 20, 30, 40, 50 and 60 min respectively for synthetic effluent. The results of decolorization for industrial effluent 1 were 57.23, 62.25, 67.45, 72.67, 79.34, and 79.34% at 10, 20, 30, 40, 50 and 60 min. Similarly, the industrial effluent 2 showed 49.45, 53.98, 58.88, 62.08, 65.12, and 65.12% decolorization at 10, 20, 30, 40, 50 and 60 min. Synthetic effluent showed maximum% decolorization (93.57%) at 50 min while industrial effluents 1 and 2 showed maximum decolorization 79.34% and 65.12% at 50 min, respectively (Figure 8). However, chemical treatment followed by biological treatment using *A. niger* gave more enhanced decolorization results for dye effluents than the individual treatments. Use of advanced oxidation after biological treatment is an effective way to degrade organic compounds, and hence to reduce or even eliminate the toxicity of textile effluents [32]. In the sequential process, the addition of an ABR reactor systems prior to conventional activated sludge systems, which are in use in many industries in Iran, resulted in 90% color removal efficiency, particularly in textile and dye industries [33]. Fenton oxidation and anoxic biological treatment, were integrated with the purpose of reducing the treatment costs via less need for oxidant and catalyst and reduced sludge production [34].

### 2.4. Mineralization Study

**Figure 9:**
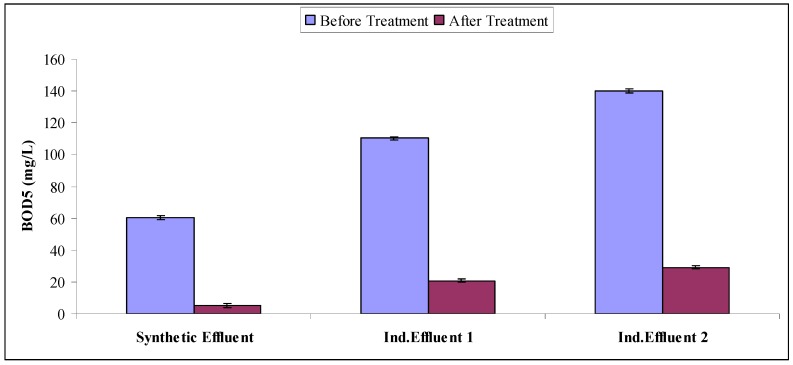
BOD_5_ of untreated and treated effluents.

**Figure 10:**
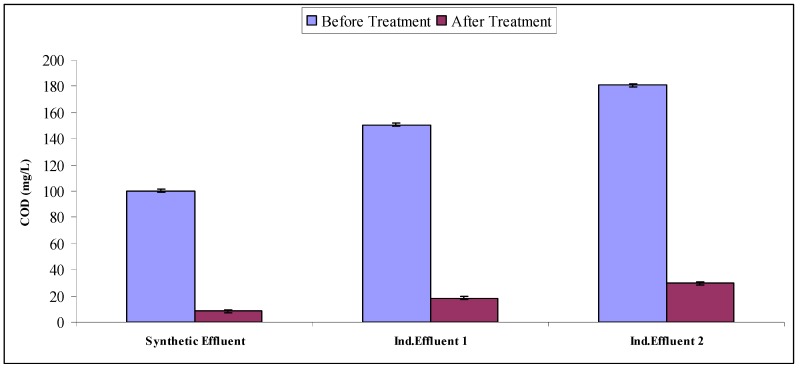
COD of intreated and treated effluents.

**Figure 11:**
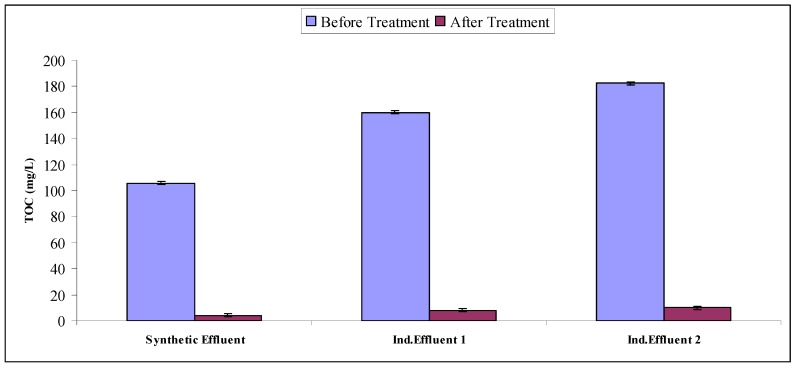
TOC of intreated and treated effluents.

Water quality parameters are a very efficient way of evaluating the mineralization potential of treated effluents. Synthetic and industrial effluents subjected to Fenton treatment followed by biological treatment using *A. niger* were then subjected to the determination of water quality parameters like to BOD, COD and TOC. All effluent treatments resulted in significant decreases in BOD, COD, TOC as compared to untreated ones (as is evident from Figure 9, Figure 10 and Figure 11). When comparing the synthetic effluent mineralization ability as compared to industrial effluents, synthetic effluent showed more decrease in water quality parameters. Researchers showed that *Phanerochaete chrysosporium* (*P. chrysosporium*) and *A. niger* reduced COD by up to 75% [35]. Researchers found a more than 90% COD reduction in a chemical treatment followed by biological treatment sequence for simulated wastewater [36]. Researchers documented an 84% reduction of TOC due to Acid Orange 24 using photo-Fenton degradation followed by biological treatment using white rot fungal cultures [37]. Our results are in agreement with previous literature reports [9,22,28].

From the current study, it can be concluded that Fenton treatment and biological treatment using *A. niger* are effective in the detoxification of contaminants like noxious dyes. However, when both treatments were used in combination better results were obtained in terms of detoxification of hazardous chemicals.

## 3. Materials and Methods

### 3.1. Chemicals

**Figure 12:**
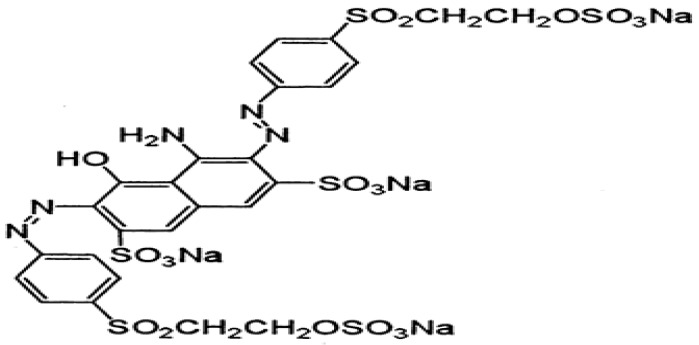
Reactive Black 15 dye.

**Figure 13:**
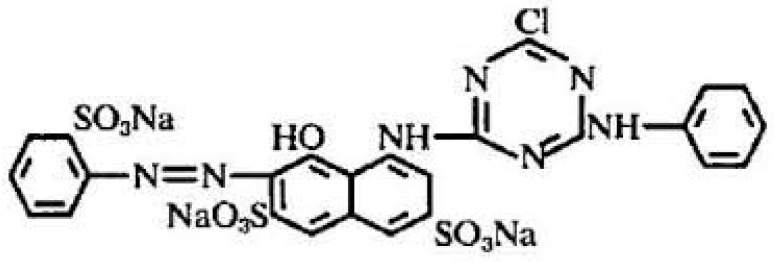
Reactive Red C-4 BL dye.

**Figure 14:**
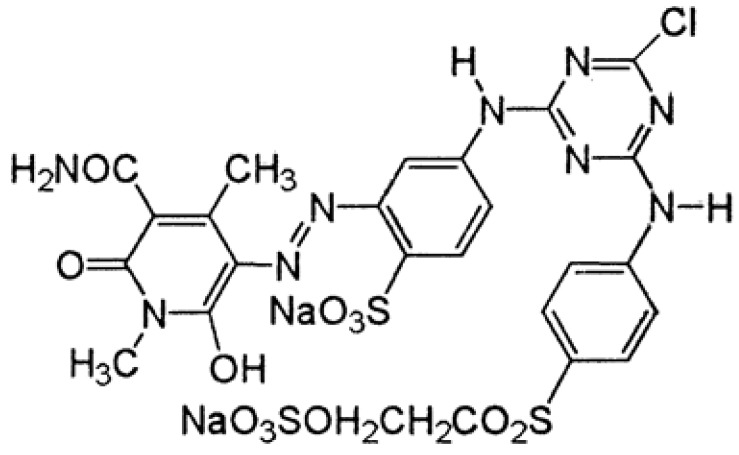
Lemon Yellow C-4 GL dye.

All chemicals were of analytical grade. Reactive dyes (the structures are given below) were purchased from a local market (Faisalabad, Pakistan). One industrial effluent (Industrial effluent 1) was collected from the Nishat Textile Industry (Faisalabad, Pakistan). The second industrial effluent (Industrial effluent 2) (Figure 12, Figure 13 and Figure 14) was collected from Bismillah Textiles Industry (Pvt Ltd, Faisalabad, Pakistan).

### 3.2. Preparation of Synthetic Effluent

A synthetic dyes wastewater was prepared by mixing hydrolysed reactive dyes (Reactive Black 15 (139 mg/L); Reactive Yellow C-4 GL (150 mg/L); Reactive Red C-4 BL (1215 mg/L), hydrolysed starch (13.9 mg/L), Na_2_SO_4_ (27.8 mg/L) and Na_2_HPO_4_ (27.8 mg/L)) in deionized water. Then it was placed on a hot plate equipped with a magnetic stirrer at 80 °C for 1.5 h after adjustment to pH 12.

### 3.3. Isolation of Microorganisms

Soil samples were collected from different local sites and were stored in sterilized polythene bags. Ten g of soil was dissolved in 100 mL of sterilized distilled water. The soil samples were homogenized by placing flasks containing the soil samples on a rotatory shaker for a period of 15 min. Different dilutions (10^−3^ to 10^−7^) were made from this soil stock solution under aseptic conditions. One mL of each dilution was transferred to Petri plates containing potato dextrose agar media (PDA media) at pH 4.5. The Petri plates were placed in an incubator for 4–5 days at 30 °C. After that, the colonies of *A. niger* were picked and transferred to PDA slants at pH 4.5. The PDA slants were incubated at 30–35 °C for a period of one week for maximum sporulation. The PDA slants having *A. niger* colonies were stored at 4 °C in a refrigerator till further usage.

### 3.4. Inoculum Preparation

The flasks containing PDA medium (whose composition was mentioned earlier) for individual fungi were adjusted at pH 4.5 with 1.0 M NaOH/1.0 M HCl and autoclaved at 121 °C for 15 min. The flasks were inoculated with loopfuls of fungal spores from individual slant cultures and placed in an incubator under shaking (120 rpm) at 30 °C for the days needed to get a homogenous spore suspension. The number of spores in the inoculum was counted by using a heamocytometer to get 10^−7^–10^−8^ spores/mL. Fresh inoculums were prepared for each experiment [38].

### 3.5. Morphological Characterization of Isolated A. niger

The macroscopic characteristics such as colony features, growth rate, and pigmentation of the isolated *A. niger* were observed and noted. Isolated colonies were also cultured on plates having PDA media for fungal sporulation. Slide preparations of fungal isolates were prepared, stained with Lactophenol Cotton Blue, viewed microscopically at 400–1000× and compared to manual data [39].

### 3.6. Experimental Procedure for Fenton Treatment

Seventy mL of each solution (i.e., synthetic effluent, H_2_O_2_ and FeSO_4_ solution) was placed in a reaction vessel. The initial pH of the sample was carefully adjusted to 3.5 by pH meter using 0.5 M H_2_SO_4_/1 M NaOH. The sample was stirred on a magnetic stirrer and the reaction was allow to run for one hour at 50 °C. Absorbance of samples was calculated every 10 min at a λ_max_ of 612 nm.

### 3.7. Experimental Procedure for Biological Treatment

One hundred mL of synthetic textile dye solution and 100 mL of Kirk′s medium were taken in flasks and after adjusting the pH at 4.5, the flasks were autoclaved for 15 min at 121 °C. After cooling to room temperature, the triplicate dye solution containing flasks were inoculated with 5 mL homogeneous spores inocula of the respective fungi and incubated at 30 °C for a period of five days. Supernatants obtained after centrifugation (1200 rpm for 10 min) were run through spectrophotometer to check the absorbance at a λ_max_ of 612 nm. The results obtained were recorded as the average of three replicates of various intervals. Samples having all nutrients except fungi were taken as abiotic controls. The decolorization (%) was measured after every 24 h for a period up to 96 h. A two-stage sequential Fenton′s oxidation followed by aerobic biological treatment using *A. niger* was used to achieve decolorization and to enhance mineralization effluents under study.

### 3.8. Decolorization Assay via UV-Vis Spectroscopy

The decolorization (%) efficiency of the parameters was assessed by using absorbance of UV/Visible spectrophotometer. The following formula was used to calculate decolorization (%) using the following formula:

Decolorization % = (I − F)/I × 100(1)

where I = initial absorbance and F = Absorbance of decolorized medium.

### 3.9. Enzyme Studies

#### 3.9.1. Lignin Peroxidase Assay

One mL of veratryl alcohol (1 mM) and 1 mL of tartrate buffer (1 mM) of pH 3 was added to 100 µL of culture supernatant. H_2_O_2_ (500 µL, 0.2 mM) was added as an oxidizing agent. The oxidation rate of veratryl alcohol to veratraldehyde was determined at 310 nm (molar extinction coefficient = 9300 M^−1^·cm^−1^).

#### 3.9.2. Manganese Peroxidase Assay

A mixture containing 1 mL of MnSO_4_ (1 mM) and 1 mL of sodium malonate buffer of pH 4.5 (50 mM) was added to 100 µL of culture supernatant. H_2_O_2_ (500 µL, 0.1 mM) was added as an oxidizing agent. The oxidation rate was monitored at 270 nm (molar extinction coefficient = 11,590 M^−1^·cm^−1^).

#### 3.9.3. Laccase Assay

A mixture containing 1 mL of ABTS (1 mM) and 1 mL of malonate buffer of pH 4.5 (50 mM) was added to 100 µL of culture supernatant. H_2_O_2_ (500 µL, 0.2 mM) was added as an oxidizing agent. Laccase activity was determined by monitoring the oxidation of 2,2-azinibis (3-ethylbenzothiazoline-6-sulfonic acid) (ABTS) at 436 nm (molar extinction coefficient = 36,000 M^−1^·cm^−1^) [40,41,42].

### 3.10. Mineralization Study

The mineralization study was carried out by measuring the water quality assurance parameters like BOD, COD, TOC following standard protocols [43].

## References

[1] Pandey A., Singh P., Iyengar L. (2007). Bacterial decolorization and degradation of azo dyes. Int. Biodeterior. Biodegrad..

[2] Kiran S., Ali S., Asgher M. (2013). Degradation and mineralization of azo dye reactive blue 222 by sequential photo-Fenton′s oxidation followed by aerobic biological treatment using white rot fungi. Bull. Environ. Contam. Toxicol..

[3] Pinheiro H.A. (2004). Physiological and Morphological Adaptations as Associated with Drought Tolerance in Robusta Coffee (*Coffea canephora* Pierre var. *kouillou*). Ph.D. Thesis.

[4] Gogate P.R., Pandit A.B. (2004). A Review of Imperative Technologies for Wastewater Treatment I: Oxidation Technologies at Ambient Conditions. Adv. Environ. Res..

[5] Hao O.J., Kim H., Chang P.C. (2000). Decolorization of wastewater. Crit. Rev. Environ. Sci. Technol..

[6] Gautam M., Azmi W. (2017). Purification of extracellular collagenase from *Pseudomonas* sp. remarkable collagenolytic activity. Adv. Biotechnol. Microbiol..

[7] Guilard C., Disdier J., Monnet C., Dussaud J., Malato S., Aldonado B., Herrmann J.M. (2003). Suspensions using artificial and solar light: Intermediates and degradation pathways. Appl. Catal. B. Environ..

[8] Ajao A.T., Adebayo G.B., Yakubu S.E. (2017). Bioremediation of textile industrial effluent using mixed culture of *Pseudomonas aeruginosa* and *Bacillus subtilis* immobilized on agar agar in a bioreactor. J. Microbiol. Biotechnol. Res..

[9] Joshi P.K., Swarup A., Maheshwari S., Kumar R., Singh N. (2011). Bioremediation of heavy metals in liquid media through fungi isolated from contaminated sources. Ind. J. Microbial..

[10] Fu Y., Viraraghavan T. (2001). Fungal decolorization of dye wastewaters. A review. Bioresour. Technol..

[11] Guo W., Yang Z., Zhou X.J., Wu Q. Degradation and mineralization of dyes with advanced oxidation processes (AOPs): A brief review. Proceedings of the 2015 International Forum on Energy, Environment Science and Materials.

[12] Sun X., Wang C., Li Y., Wang W., We J. (2015). Treatment of phenolic wastewater by combined UF and NF/RO processes. Desalination.

[13] Chatha S.A.S., Kiran S., Gulzar T., Kamal S., Ghaffar A., Chatha M.N. (2016). Comparative study on decolorisation and mineralisation of synthetic and real textile effluents using advanced oxidation processes. Oxid. Commun..

[14] Rashid A., Nosheen S., Kiran S., Bhatti H.N., Kamal S., Shamim F., Rafique M.A. (2016). Combination of oxidation and coagulation processes for wastewater decontamination on batch scale. Oxid. Commun..

[15] Jain B., Singh A.K., Sharma V.K. (2017). Degradation of naphthylazo anionic dye by Fenton and Fenton-like processes: a comparative study with Fast Sulphon Black-F. Desalin. Water Treat..

[16] Ali N.F., El-Mohamedy R.S.R. (2012). Microbial decolourization of textile waste water. J. Saudi Chem. Soc..

[17] Omar S.A. (2016). Decolorization of Different Textile Dyes by Isolated *Aspergillus niger*. J. Environ. Sci. Technol..

[18] Bergsten-Torralba L.R., Nishikawa M.M., Baptista D.F., Magalhaes D.P., Silva M.D. (2009). Decolorization of different textile dyes by *Penicillium simplicissimum* and toxicity evaluation after fungal treatment. Braz. J. Microbiol..

[19] Rana R.S., Singh P., Kandari V., Singh R., Dobhal R., Gupta S. (2017). A review on characterization and bioremediation of pharmaceutical industries′ wastewater: An Indian perspective. Appl. Water Sci..

[20] Punzi M., Anbalagan A., Börner R.A., Svensson B.M., Jonstrup M., Mattiasson B. (2015). Degradation of a textile azo dye using biological treatment followed by photo-Fenton oxidation: Evaluation of toxicity and microbial community structure. Chem. Eng. J..

[21] Ilyas S., Rehman A. (2013). Decolorization and detoxification of Synozol red HF-6BN azo dye by *Aspergillus niger* and *Nigrospora *sp. J. Environ. Health Sci. Eng..

[22] Taskin M., Serkan E. (2010). Reactive dye bioaccumulation by fungus *Aspergillus niger* isolated from the effluent of sugar fabric-contaminated soil. Toxicol. Ind. Health.

[23] Bahmani S., Raj B., Boufounos P.T. (2013). Greedy sparsity-constrained optimization. J. Mach. Learn. Res..

[24] Deepika R., Sathyabama N., Sankareswaran M., Anbalagan S., Vinayaga M.D., Kamalakkannan V. (2014). Bioremediation of textile effluent with *Aspergillus niger*-based silver nanoparticles and its field trial. J. Environ. Sci. Comput. Sci. Eng. Technol..

[25] Rani B., Kumar V., Singh J., Bisht S., Teotia P., Sharma S., Kela R. (2014). Bioremediation of dyes by fungi isolated from contaminated dye effluent sites for bio-usability. Braz. J. Microbiol..

[26] Ali H., Ahmed W., Haq T. (2009). Decolorization and degradation of malachite green by *Aspergillus flavus* and *Alternaria solani*. Afr. J. Biotechnol..

[27] Sharma P., Jha A.B., Dubey R.S., Pessarakli M. Reactive oxygen species, oxidative damage, and antioxidative defense mechanism in plants under stressful conditions. J. Bot..

[28] Kiran S., Ali S., Asgher M., Anwar F. (2012). Comparative study on decolorization of Reactive Dye 222 by white rot fungi *Pleurotus ostreatus* IBL-02 and *Phanerochaete chrysosporium* IBL-03. Afr. J. Microbiol. Res..

[29] Waghmode T.R., Kurade M.B., Khandare R.V., Govindwar S.P. (2011). A sequential aerobic/microaerophilic decolorization of sulfonated monoazo dye Golden Yellow HER by microbial consortium GG-BL. Int. Biodeterior. Biodegrad..

[30] Waghmode T.R., Kurade M.B., Lade H.S., Govindwar S.P. (2012). Decolorization and biodegradation of Rubine GFL by microbial consortium GG-BL in sequential aerobic/microaerophilic process. App. Biochem. Biotechnol..

[31] Lade H., Kadam A., Paul D., Govindwar S. (2015). Biodegradation and detoxification of textile azo dyes by bacterial consortium under sequential microaerophilic/aerobic processes. EXCLI J..

[32] Meerbergen K., Crauwels S., Willems K.A., Dewil R., Van-Impe J., Appels L., Lievens B. (2017). Decolorization of reactive azo dyes using a sequential chemical and activated sludge treatment. J. Biosci. Bioengng..

[33] Naimabadi A., Attar H.M., Shahsavani A. (2009). Decolorization and biological degradation of azo dye Reactive Red 2 by anaerobic/aerobic sequential process. J. Environ. Health Sci. Eng..

[34] Bahmani P., Kalantary R.R., Esrafili A., Gholami M., Jafari A.J. (2013). Evaluation of Fenton oxidation process coupled with biological treatment for the removal of Reactive Black 5 from aqueous solution. J. Environ. Health Sci. Eng..

[35] Singh S., Pakshirajan K. (2010). Enzyme activities and decolourization of single and mixed azo dyes by the white-rot fungus *Phanerochaete chrysosporium*. Int. Biodeterior. Biodegrad.

[36] Kiran S., Ali S., Asgher M., Shahid S.A. (2012). Photo-fenton process: Optimization and decolourization and mineralization of Reactive Blue 222 dye. J. Environ. Sci. Water Resour..

[37] Naseer A., Nosheen S., Kiran S., Kamal S., Javaid M.A., Mustafa M., Tahir A. (2016). Degradation and detoxification of Navy Blue CBF dye by native bacterial communities: an environmental bioremedial approach. Desalin. Water Treat..

[38] Kiran S., Adeel S., Nosheen S., Hassan A., Usman M., Rafique M.A. (2017). Recent trends in textile effluent treatments: A review. Adv. Mater. Wastewater Treat..

[39] Larone DH. (2002). Medically Important Fungi: A Guide to Identification.

[40] Qin X., Sun X., Huang H., Bai Y., Wang Y., Luo H., Su X. (2017). Oxidation of a non-phenolic lignin model compound by two *Irpex lacteus* manganese peroxidases: Evidence for implication of carboxylate and radicals. Biotechnol. Biofuels.

[41] Mishra B., Kumbhare L.B., Jain V.K., Priyadarsini K.I. (2008). Pulse radiolysis studies on reactions of hydroxyl radicals with selenocystine derivatives. J. Phys. Chem. B.

[42] Datta R., Kelkar A., Baraniya D., Molaei A., Moulick A., Meena R.S., Formanek P. (2017). Enzymatic degradation of lignin in soil: A review. Sustainability.

[43] Greenberg A.E., Trussel R.L., Clesceri L.S. (1985). Standard Methods for the Examination of Water and Wastewater.

